# Characterization of gut microbiota profile in Iranian patients with bipolar disorder compared to healthy controls

**DOI:** 10.3389/fcimb.2023.1233687

**Published:** 2023-09-21

**Authors:** Nassir Rashnaei, Abbas Akhavan Sepahi, Seyed Davar Siadat, Esmaeil Shahsavand-Ananloo, Golnaz Bahramali

**Affiliations:** ^1^ Department of Microbiology, Faculty of Biological Sciences, Islamic Azad University, North Tehran Branch, Tehran, Iran; ^2^ Department of Mycobacteriology and Pulmonary Research, Pasteur Institute of Iran, Tehran, Iran; ^3^ Department of Psychosomatic, Imam Khomeini Hospital Complex, School of Medicine, Tehran University of Medical Sciences (TUMS), Tehran, Iran; ^4^ Hepatitis and AIDS Department, Pasteur Institute of Iran, Tehran, Iran

**Keywords:** bipolar disorder, gut-brain-axis, gut microbiota, mental disorder, qPCR assessment

## Abstract

**Introduction:**

The human gut microbiota plays a crucial role in mental health through the gut-brain axis, impacting central nervous system functions, behavior, mood, and anxiety. Consequently, it is implicated in the development of neuropsychiatric disorders. This study aimed to assess and compare the gut microbiota profiles and populations of individuals with bipolar disorder and healthy individuals in Iran.

**Methods:**

Fecal samples were collected from 60 participants, including 30 bipolar patients (BPs) and 30 healthy controls (HCs), following rigorous entry criteria. Real-time quantitative PCR was utilized to evaluate the abundance of 10 bacterial genera/species and five bacterial phyla.

**Results:**

Notably, *Actinobacteria* and *Lactobacillus* exhibited the greatest fold change in BPs compared to HCs at the phylum and genus level, respectively, among the bacteria with significant population differences. *Ruminococcus* emerged as the most abundant genus in both groups, while *Proteobacteria* and *Bacteroidetes* showed the highest abundance in BPs and HCs, respectively, at the phylum level. Importantly, our investigation revealed a lower *Firmicutes/Bacteroidetes* ratio, potentially serving as a health indicator, in HCs compared to BPs.

**Conclusion:**

This study marks the first examination of an Iranian population and provides compelling evidence of significant differences in gut microbiota composition between BPs and HCs, suggesting a potential link between brain functions and the gut microbial profile and population.

## 1 Introduction

Psychiatric disorders are behavioral or mental disorders, which cause discomfort or disruption in personal, occupational, and social activities and have significant consequences for the patient’s life (Bolton, 2013). The life expectancy of individuals with these disorders is one to two decades shorter than that of healthy people (Hennekens et al., 2005; Kupfer, 2005; Nordentoft et al., 2013; Lomholt et al., 2019). Studies have shown that the prevalence of psychiatric disorders varies in different countries, depending on factors, such as welfare, level of education, and income (Demyttenaere et al., 2004).

According to the World Health Organization (WHO), one in four people experience at least one mental disorder in their lifetime, with bipolar disorder (BD) being one of the most severe ones (Demily et al., 2009; Holtz, 2017; Painold et al., 2019). A systematic review conducted in 2007 showed that the prevalence of mental disorders is 29% in Iran, which is almost 11.4% higher than the global rate (Taheri Mirghaed et al., 2020). The WHO also reported that the lifetime prevalence of BD is 1% to 2% around the world. Generally, BD is recognized as a type of mood disorder, characterized by neurological and psychiatric problems and immune and physiological imbalances (Marwaha et al., 2013; Kaplan et al., 2014). Bipolar Disorders (BD) are now recognized as a multidimensional and intricate condition, influenced by a myriad of factors, encompassing age-related physiological alterations, substance misuse, temperament traits, and susceptibility to stressful life events, as substantiated by their notable associations with alcohol and drug dependence, alongside cigarette smoking (Wilens et al., 2008; Chauvet-Gélinier et al., 2012; Mutz et al., 2022).

The prevalence of mental disorders is high in different countries, and the significant cost of rehabilitation and treatment is a major challenge (Christensen et al., 2020). Following the COVID-19 pandemic, the prevalence of these disorders has increased significantly in recent years, emphasizing the importance of these disorders (Chekole and Abate, 2021; Khademi et al., 2021; Li Y.et al., 2022). There have been persistent efforts to find new ways to diagnose and treat BD, and several methods have been proposed (Culpepper, 2014). However, this disorder is still classified and recognized by its phenotypic characteristics, and there is no verified biomarker to distinguish bipolar patients (BPs) from healthy individuals (Chen et al.; Lichtenstein et al., 2009; Van Snellenberg and De Candia, 2009).

Recently, considerable attention has been paid to the analysis of human microbiota as a promising biomarker to diagnose and treat BD (Dickerson et al., 2017). The gut microbiota imbalance is associated with metabolic disorders (e.g., diabetes and obesity), irritable bowel syndrome, celiac disease, gastrointestinal inflammatory disease, and neurodegenerative diseases (Sampson et al., 2016; Rodrigues-Amorim et al., 2018). Bowel and digestive disorders, including irritable bowel syndrome, colitis, and celiac disease, are also prevalent complications in people with mental disorders (Severance et al., 2015). Additionally, digestive disorders are the third leading cause of mortality in schizophrenia patients, which is also known as the gastrointestinal diseases (Saha et al., 2007). Also, gastrointestinal diseases are common in BPs, which can be related to the gut microbial profile (Flowers et al., 2020). Along with the intensity and frequency of gastrointestinal symptoms, depression And anxiety symptoms increase in humans (Smith and Wissel, 2019).

Recent studies have shown that the gut-brain axis establishes a bidirectional relationship between the gastrointestinal microbiota and the brain and significantly affects the brain function and host behaviors (Collins et al., 2012; Mangiola et al., 2016; Kang et al., 2017; Knuesel and Mohajeri, 2021; Góralczyk-Bińkowska et al., 2022). Nevertheless, the exact mechanism of the effect of gastrointestinal microorganisms on the brain function and mental disorders, such as BD, is not fully understood, although it seems to involve the vagus nerve, hormones, immune system, tryptophan metabolism, and microbial metabolites, such as short-chain fatty acids (SCFAs) (Round and Mazmanian, 2009; Cryan and Dinan, 2012; Kamada et al., 2013; Dinan and Cryan, 2015; Góralczyk-Bińkowska et al., 2022). For instance, 90-95% of serotonin in the body is produced by the gut bacteria (Terry and Margolis, 2017; Strandwitz, 2018). Serotonin, as a neurotransmitter mediating happiness and well-being, has significant effects on mental disorders, especially BD. This hormone is known to decrease in the depressive phase, but is controversial in the manic phase (Mahmood and Silverstone, 2001). Evidence suggests that some *Proteobacteria*, *Lactobacillus* and *Streptococcus* species are serotonin-producing bacteria (Galland, 2014; Clark and Mach, 2016; Mazzoli and Pessione, 2016; Kaur et al., 2019).

SCFAs are metabolites produced by bacteria, which play a significant positive role in intestinal inflammation and human health. The importance of these metabolites has been confirmed in BPs (Silva et al., 2020; McGuinness et al., 2022). The phyla including *Proteobacteria*, *Actinobacteria, Bacteroidetes, Verrucomicrobia* and *Firmicutes*, genera including *Bifidobacterium*, *Clostridium*, *Dialister*, *Lactobacillus*, *Ruminococcus*, and *Streptococcus*, and species including *Akkermansia muciniphila*, *Coprococcus comes*, and *Faecalibacterium prausnitzii* have been shown to produce SCFAs, which can be exchanged with the host human blood (Ríos-Covián et al., 2016; Markowiak-Kopeć and Śliżewska, 2020; Akhtar et al., 2022; Houtman et al., 2022).

Dopamine, a neurotransmitter, plays a pivotal role in regulating mood, motivation, reward, and pleasure (Bromberg-Martin et al., 2010). In bipolar disorder, there is evidence of anomalous dopamine activity in the brain that potentially exacerbates the symptoms of the disorder (Ashok et al., 2017). Some gut bacteria can affect dopamine metabolism and function, including *Lactobacillus*, *Bifidobacterium*, *Ruminococcus*, *Clostridium*, and *Enterococcus faecalis* (González-Arancibia et al., 2019; Hamamah et al., 2022).

Additionally, some gut microbiota bacteria, including *Enterococcus faecalis*, *proteobacteria, Bifidobacterium*, *Streptococcus*, and *Lactobacillus*, are capable of producing gamma-aminobutyric acid (GABA), as a major inhibitory neurotransmitter which plays a key role against anxiety and depressive disorders in mammals (Dhakal et al., 2012; Dagorn et al., 2013; Duranti et al., 2020). Besides, the *Firmicutes*/*Bacteroidetes* (F/B) ratio plays a vital role in intestinal homeostasis, as it is directly related to the level of health, and its disruption leads to dysbiosis (Mariat et al., 2009; Magne et al., 2020; Stojanov et al., 2020).

This study aimed to measure and compare the abundance of 10 genera/species and five phyla bacteria in the fecal samples of BPs versus healthy controls (HCs) who had no history of mental disorders. Regarding the ever-increasing information about the relationship between the brain and the gut microbiota, novel studies are underway to identify the pathways of this interaction; such information can help us discover new methods of diagnosis and treatment for BD. In line with these efforts, it seems essential to find a specific bacterial biomarker for diagnostic purposes.

## 2 Methods

### 2.1 Study population

All participants in this study were selected from Roozbeh Psychiatric Hospital and the Psychosomatic Ward of Imam Khomeini Hospital (Tehran, Iran). They first completed an informed consent form and then, a prepared questionnaire. Initially, 54 people with presumed symptoms of bipolar disorders were evaluated. Among them, 30 people were recognized as BPs and participated in sample collection. To provide equal number of healthy individuals as controls, 39 apparent healthy people contributed to answering questionnaire out of which 30 people considered as HCs and donated the required samples. Therefore, a total of 60 BP and HC people who met the inclusion criteria were examined and the rest were excluded. The inclusion criteria were as follows: age 18–65 years old, BMI 18–35 kg/m^2^, no history of bowel or gastric surgery or history of specific diseases, absence of alcohol or antibiotic use in the past three months, absence of acute infectious diseases, absence of regular consumption of prebiotics or probiotics, not current pregnancy or breastfeeding, and no adherence to special diets and Patients without intellectual disability. The BP group included individuals with symptoms of BD, confirmed by two psychologists according to the Diagnostic and Statistical Manual of Mental Disorders, Fifth Edition (DSM-5), regardless of the disorder phase, whereas the HC group included the volunteer personnel of the abovementioned hospitals, who were confirmed to be healthy by the same psychologist. It is worth mentioning that many people with bipolar symptoms refused contribution to our study often due to their special mental situations. The patients were not requested to provide an explanation for their decision to decline participation in this study. Nevertheless, a few individuals voluntarily expressed their pessimism or concerns regarding its safety. This was one of the main reasons that limited our samples sizes. The present study was approved by the Iran National Committee for Ethics in Biomedical Research with ethics code IR.IAU.TNB.REC.1400.119. Informed consent was obtained from all participants before enrollment in the study.

### 2.2 Blood of clinical characteristics

Blood samples were collected from all participants included in this study in morning concurrent with stool sample. A volume of 10 milliliters of blood was procured from each person and expeditiously transferred to the Pasteur diagnostic laboratory for analyzing the blood parameters including FT3, FT4, TSH, 25 hydroxy vitamin D3, Cholesterol, Triglyceride, Na, K, FBS, Urea, Creatinine, AST, ALT, Phosphorus, Calcium. The parameters were measured using standard laboratory methods. FT3, FT4, TSH and D3 levels were quantified through immunoassays, employing enzyme-linked immunosorbent assays (ELISA). Cholesterol and Triglyceride levels were measured by enzymatic colorimetric. Na and K were determined using ion-selective electrode (ISE). FBS was evaluated via the glucose oxidase method. Urea and Creatinine were quantified using colorimetric assays based on urease and Jaffe reaction, respectively. AST and ALT were measured through kinetic assays. Phosphorus and Calcium levels were determined using colorimetry.

Mean values from the BP and HC groups were compared using Student’s T-test to survey any probable clinical characteristic- or health-related differences between the two groups.

The mean values of clinical characteristics obtained from the two groups are shown in **Table 1**.

**Table 1:** Baseline demography and clinical characteristics.

	BP	HC	P_value_	Normal range
Parameters	30	30	0.611	
Gender	13 male/17 female	15 male/15 female		
Age(years)*	38.5 ± 11.68	39 ± 9.47	0.903	
Weight (kg)	73.46 ± 12.95	65.9 ± 8.12	0.252	
Height (m)	1.67 ± 0.10	1.68 ± 8.43	0.683	
BMI (kg/m2)	26.33 ± 4.29	22.11 ± 1.36	0.103	18.5-24.9
FT3 (pg/mL)	3.21 ± 0.35	4.98 ± 0.55	<0.001	2.3 - 4.2
FT4 (pg/mL)	1.317 ± 0.33	1.07 ± 0.29	0.087	0.7- 1.9
TSH (mIU/mL)	2.3 ± 1.46	2.36 ± 1.06	0.914	0.5- 5.0
25 hydroxy vitamin D3 (ng/mL)	38.34 ± 10.19	45.66 ± 21.40	0.470	20-40
Cholesterol (mg/dL)	188.75 ± 49.29	150.33 ± 34.09	0.023	Less than 200
Triglyceride (mg/dL)	150.37 ± 54.40	115.37 ± 58.28	0.170	Less than 150
Na (mEq/L)	139.5 ± 2.39	140.43 ± 1.80	0.267	135-145
K (mEq/L)	4.25 ± 0.32	4.21 ± 0.33	0.757	3.6-5.2
FBS (mg/dL)	90.37 ± 14.00	90.5 ± 9.36	0.978	60-110
Urea (mg/dL)	49.87 ± 38.18	26.67 ± 7.63	0.030	15-50
Creatinine (mg/dL)	1.48 ± 1.47	0.95 ± 0.14	0.118	0.7-1.4
AST (U/L)	24 ± 5.25	22.11 ± 8.40	0.614	8-33
ALT (U/L)	25.67 ± 20.84	21.81 ± 7.49	0.519	4-36
Phosphorus (mg/dL)	8.77 ± 0.98	9.27 ± 0.43	0.396	2.5-5
Calcium (mg/dL)	8.771 ± 0.56	9.266 ± 0.39	0.014	8.6-10.3

BP, Bipolar patient; HC, Healthy controls.

*Data are shown as means values ± sd. P_value_ < 0.05 considered as significantly different.

Abbreviations: BMI: Body Mass index; FT3: Free Triiodothyronine; FT4: Free Thyroxine; TSH: Thyroid-Stimulating Hormone; FBS: Fasting Blood Sugar; AST: Aspartate Transaminase; ALT: Alanine Transaminase.

### 2.3 Fecal sample collection and DNA extraction

Fecal samples were collected from each individual, placed in a sterile stool collection cup, and immediately transferred to laboratory on ice; they were stored at -80°C for further assessments. About 2 g of each fecal sample was used for DNA extraction with a QIAamp Power Fecal Pro DNA Kit (Germany) according to the manufacturer’s instructions. The concentration and purity of extracted DNA were determined by a NanoDrop™ spectrophotometer (Thermo Scientific, USA), while the quality of DNA was examined by 1% agarose gel electrophoresis.

### 2.4 Real-time quantitative polymerase chain reaction assay

According to various databases (https://hmdb.ca/, https://microbiomedb.org/, and https://microbiomology.org/) and numerous studies in the literature on microbiota and psychological disorders, five phyla which might contribute to BD, including *Actinobacteria*, *Bacteroidetes*, *Firmicutes*, *Proteobacteria* and *Verrucomicrobia*, alongside ten bacterial genera/species, including *Akkermansia muciniphila*, *Bifidobacterium*, *Clostridium* cluster IV, *Coprococcus comes*, *Dialister*, *Enterococcus faecalis*, *Faecalibacterium prausnitzii*, *Lactobacillus*, *Ruminococcus*, and *Streptococcus* were evaluated (Serpa et al., 2010; Pessione, 2012; Galland, 2014; Louis et al., 2014; Reichardt et al., 2014; Vital et al., 2014; Clark and Mach, 2016; Koh et al., 2016; Mazzoli and Pessione, 2016; Hu et al., 2019; Kaur et al., 2019; Lai et al., 2021; Lucidi et al., 2021; McGuinness et al., 2022). qPCR was used to assess the abundance of each bacterium in the stool samples.

Specific primers based on 16s rRNA were designed for each bacterium, as listed in **Table 2**. The universal primer sequences were as follows: forward primer, TCCTACGGGAGGCAGCAGT; and reverse primer, GGACTACCAGGGTATCTAATCCTGTT (Qian et al., 2018).

**Table 2:** List of primer sequences used for qPCR.

Microorganism		Primer sequence (5’−3’)	Refs
Part A: phylum			
***Actinobacteria***	F	CGCGGCCTATCAGCTTGTTG	(Bredholt et al., 2008)
	R	CCGTACTCCCCAGGCGGGG	
***Bacteroidetes***	F	GGAACATGTGGTTTAATTCGATGAT	(Layton et al., 2006)
	R	AGCTGACGACAACCATGCAG	
***Firmicutes***	F	GGAGYATGTGGTTTAATTCGAAGCA	(Yi et al., 2019)
	R	AGCTGACGACAACCARGCAC	
***Verrucomicrobia***	F	GAATTCTCGGTGTAGCA	(Hermann-Bank et al., 2013)
	R	GGCATTGTAGTACGTGTGCA	
***Proteobacteria***		CATGACGTTACCCGCAGAAGAAG	(Murri et al., 2013)
		CTCTACGAGACTCAAGCTTGC	
Part B: Genus, species			
***Akkermansia muciniphila***	F	CAGCACGTGAAGGTGGGGAC	(Schneeberger et al., 2015)
	R	CCTTGCGGTTGGCTTCAGAT	
***Bifidobacterium spp***	F	TCGCGTCYGGTGTGAAAG	(Schneeberger et al., 2015)
	R	CCACATCCAGCRTCCAC	
***Coprococcus comes***	F	GTGACCGGCGTGTAATGACG	(Kurakawa et al., 2016)
	R	CAGAGTGCCCATCCGAATTG	
***Dialister spp***	F	CGGAATTATTGGGCGTAAAG	(Sun et al., 2017)
	R	CTTTCCTCTCCGATACTCCA	
***Enterococcus faecalis***	F	ACGTGTCTTCCATCAACGCT	(Arabestani et al., 2017)
	R	ACTGCTGTATGTTTGTCTCCGA	
***Faecalibacterium prausnitzii***	F	GGAGGAAGAAGGTCTTCGG	(Fitzgerald et al., 2018)
	R	AATTCCGCCTACCTCTGCACT	
***Lactobacillus spp***	F	AGCAGTAGGGAATCTTCCA	(Alioua et al., 2016)
	R	ATTYCACCGCTACACATG	
***Ruminococcus spp***	F	GAGTGAAGTAGAGGTAAGCGGAATTC	(Weimer et al., 2008)
	R	GCCGTACTCCCCAGGTGG	
***Streptococcus spp***	F	AGTCGGTGAGGTAACCGTAAG	(Hall-Stoodley et al., 2006)
	R	AGGAGGTGATCCAACCGCA	
Part C: *Clostridium* cluster IV			
***Clostridium* IV**	F	ACAATAAGTAATCCACCTGG	(Hermann-Bank et al., 2013)
	R	CTTCCTCCGTTTTGTCAA	

The qPCR reaction mixture was prepared using 10 μL of 2X SYBR Green PCR Mix (Yekta Tajhiz Azma, Iran), 1 μL of forward primer, 1 μL of reverse primer (**Table 2**), 2 μL of sample DNA, and 6 μL of sterile deionized water. A Roche LightCycler 480 System (Switzerland) was also used to assess the DNA concentration of different bacterial populations in the samples.

Universal primer replication was carried out to confirm the DNA and presence of bacteria and also to confirm the steps taken so far. The concentration of target bacteria was calibrated using serial dilutions of *Escherichia coli* as a standard bacterium. Next, the dsDNA copy number of each bacterium was calculated by normalizing the bacterial concentration based on the genome size. Finally, the fold change of each bacterial population was calculated in BPs relative to HCs, based on the dsDNA copy numbers. Also, the F/B ratio was calculated in both groups as an index of health status.

### 2.5 Metabolic network and correlation analysis

We conducted a literate review and summarized the bacteria that were assessed in this study for their involvement in the production of SCFA, GABA, dopamine and serotonin. These substances are commonly associated with BD. Subsequently, we constructed a hypothetical metabolic network of the bacteria. Also, we conducted a correlation analysis to demonstrate the relationship between the population of different bacteria within the experimental groups.

### 2.6 Statistical analyses

The collected data were analyzed using SPSS Version 26. T-test and independent-samples Mann-Whitney U test were used to compare the mean values of different groups. The relationships between the population of the bacteria were measured using Pearson correlation coefficient. A *P*-value less than 0.05 was considered as statistically significant.

## 3 Results

### 3.1 Gut microbiota

As shown in **Table 1**, all parameters assessed in the BP and HC groups were in the normal ranges (P>0.1). Also, all the parameters except FT3 were not significantly different between the groups. The level of free triiodothyronine (FT3) was significantly lower in BPs compared to HCs (P<0.001).

Additionally, the abundance of all bacteria assessed in this study significantly changed in BPs compared to HCs (**Table 3**).

**Table 3:** Fold change differences in the gut bacteria of BDs compared to HCs.

	BD	HC	Fold change	(P_value_)
***Actinobacteria***	2.21E-03	1.11E-05	2.00E+02	< 0.001
***Akkermansia muciniphila***	34093.05	4.23E+12	8.06E-09	< 0.001
***Bacteroidetes***	83.32341	3.58E+10	2.33E-09	< 0.001
***Bifidobacterium spp***	1492871	1.05E+13	1.42E-07	< 0.001
***Clostridium iv***	7.612204	0.086185	8.83E+01	< 0.001
***Coprococcus comes***	1.17E+05	1.29E+04	9.11E+00	< 0.001
***Dialister spp***	7031724	5.03E+09	1.40E-03	< 0.001
***Enterococcus faecalis***	1.60E+03	8.60E+01	1.86E+01	< 0.001
***Faecalibacterium prausnitzii***	8.11E+06	6.49E+11	1.25E-05	< 0.001
***Firmicutes***	400.1563	2.55E+08	1.57E-06	< 0.001
***Lactobacillus spp***	48941432	1071.715	4.57E+04	< 0.001
***Proteobacteria***	548.176	391.1138	1.40E+00	< 0.001
***Ruminococcus spp***	1.00E+08	1.16E+13	8.66E-06	< 0.001
***Streptococcus spp***	1.29E+07	2.73E+09	4.73E-03	< 0.001

BD, Bipolar disorder; HCs, Healthy controls.

The phylum *Actinobacteria* with a fold change of 200 and the genus *Lactobacillus* with a fold change of 45,700 showed the greatest changes, while the phylum *Bacteroidetes* with a fold change of 0.00000000233 and *Akkermansia muciniphila* with a fold change of 0.00000000806 showed the least changes in BPs compared to HCs. The abundance of genera/species, including *Akkermansia muciniphila*, *Bifidobacterium*, *Faecalibacterium prausnitzii*, *Dialister*, *Streptococcus*, and *Ruminococcus*, as well as phyla including *Bacteroidetes*, *Firmicutes*, and *Verrucomicrobia*, was lower in BPs, while the abundance of *Clostridium* cluster IV, *Lactobacillus, Coprococcus comes* and *Enterococcus faecalis* species, and the phylum *Actinobacteria* and *Proteobacteria* was higher in BPs compared to HCs.

**Figure 1:**
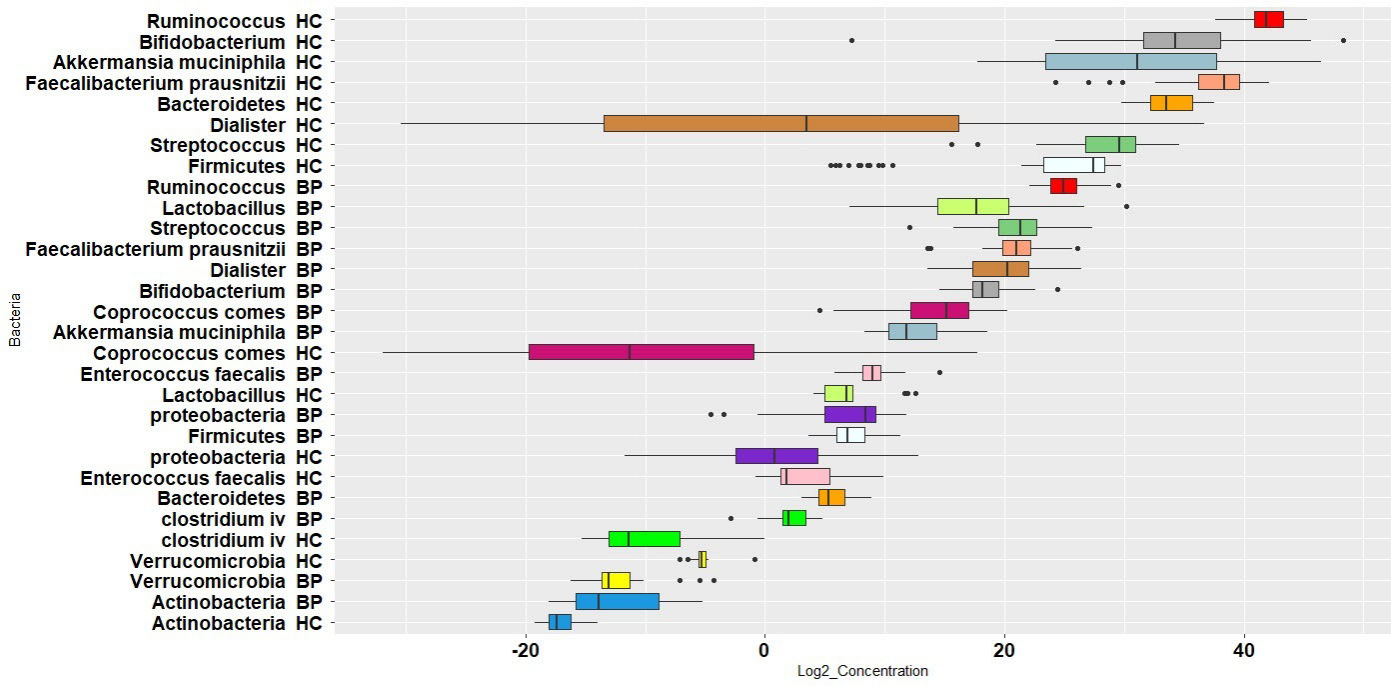
Boxplot of log 10 bacterial CFU/g stool in bipolar patients (BP) and healthy controls (HC). The population of each bacterium in the two groups is shown by same colors.

**Figure 2:**
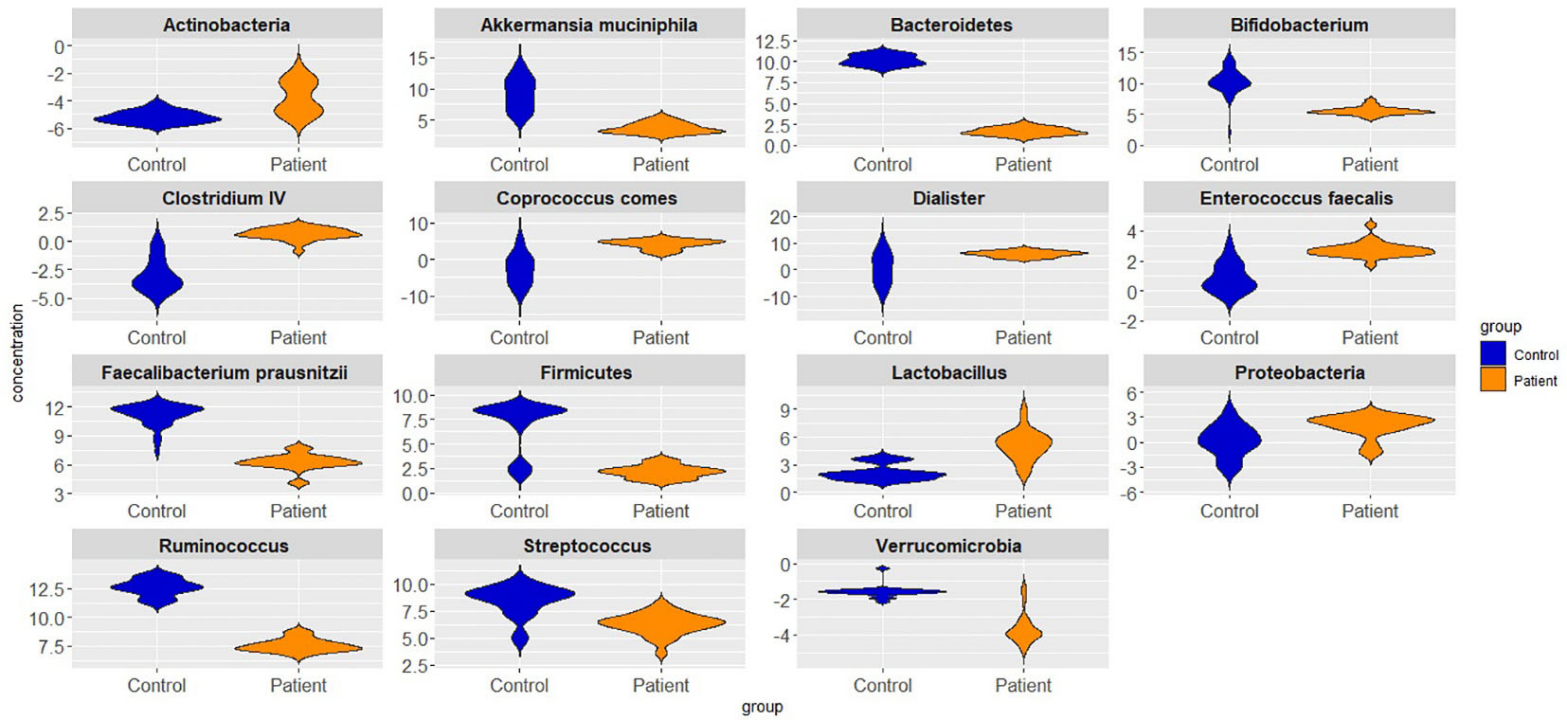
Violin plots showing different bacterial abundances in bipolar patients and healthy controls.

**Figure 1** presents log 10 CFU/g of bacteria in BP and HC groups. *Clostridium* cluster IV and the phylum *Actinobacteria* showed the lowest abundance, while the genus *Ruminococcus* and the phylum *proteobacteria* showed the highest abundance in BPs. In HCs, *Clostridium* cluster IV and the phylum *Actinobacteria* had the lowest abundance, whereas the genus *Ruminococcus* and the phylum *Bacteroidetes* were the largest populations. The concentration distribution of each bacterium in the two groups is shown in **Figure 2** for a simpler comparison.

**Figure 3:**
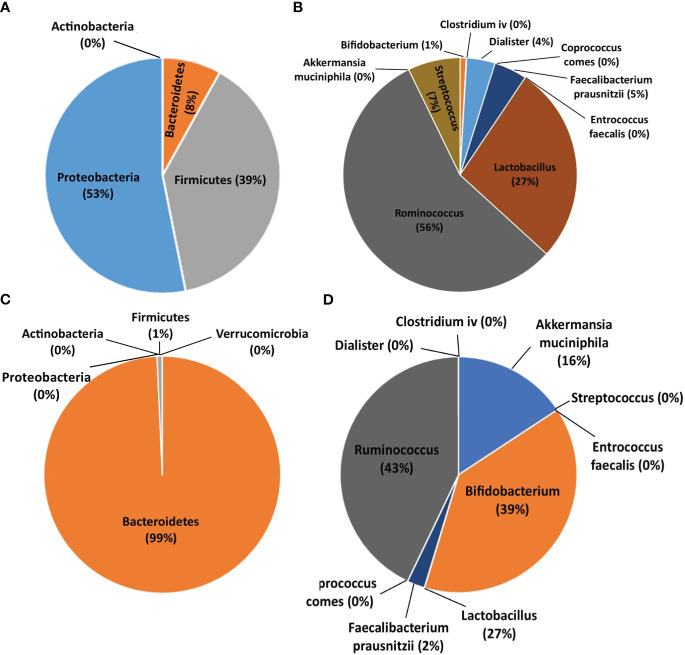
Pie charts showing the population percentage of each bacterium in bipolar patients (BPs) and healthy controls (HCs). **(A)** Abundance of bacteria at the phylum level in BPs. **(B)** Abundance of bacteria at the genus and species level in BPs. **(C)** Abundance of bacteria at the phylum level in HCs. **(D)** Abundance of bacteria at the genus and species level in HCs.

**Figure 3** summarizes the population percentage of different bacterial populations in BP and HC groups. The phylum *Proteobacteria* accounted for 53% of bacteria in BPs, while in HCs, only 1% of bacteria were in the phylum *Firmicutes*; the phylum *Bacteroidetes* comprised 99% of the assessed bacteria in HCs. On the other hand, the genus *Ruminococcus* was the predominant population in BP and HC groups (56% and 43%, respectively).

The mean concentrations of *Bacteroidetes* and *Firmicutes*, along with the F/B ratio, are presented in **Table 4**. The mean of F/B ratios was significantly higher in BPs compared to HCs.

**Table 4:** Firmicutes/Bacteroidetes (F/B) ratio.

Species	Group	(Mean ± SD)	F	(*P* value)
**F/B**	BPs	5.841 ± 6.239	44.05	<0.001
	HCs	0.018 ± 0.033		

BPs, Bipolar patients; HCs, Healthy controls; F/B, Firmicutes/Bacteroidetes.

**Figure 4:**
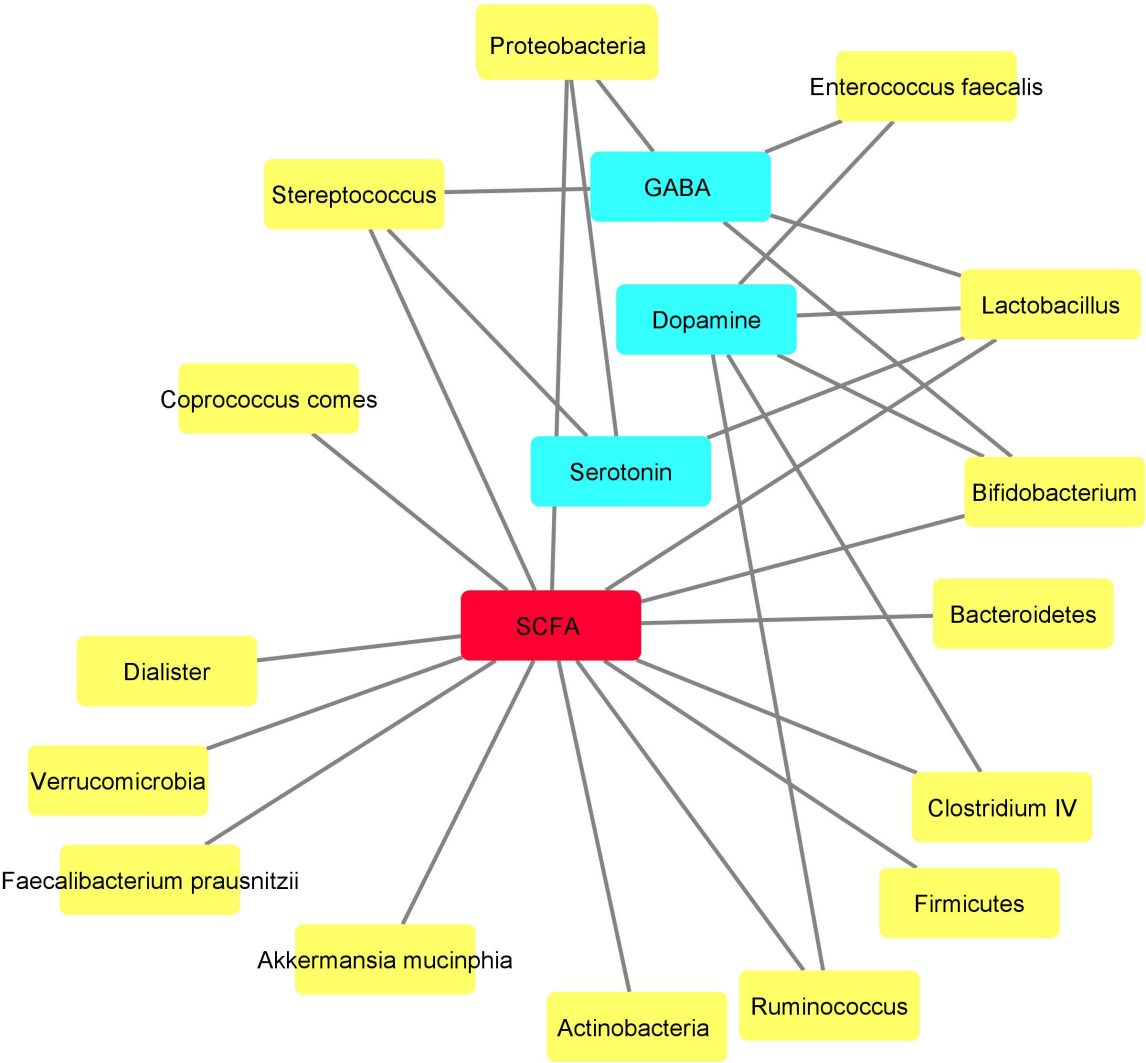
Metabolic network drawn based on the metabolites produced by the assessed bacteria using Cytoscape. Yellow and blue nodes demonstrate the bacteria and their metabolites, respectively. Short-chain fatty acid (SCFA), shown by red node, seems to function as the central metabolite in this network produced by various bacteria.

The metabolic network of the assessed bacteria is shown in **Figure 4**. Fourteen out of fifteen bacteria were SCFA-producing bacteria. Five out of these Fourteen bacterial populations could also produce other substances concomitantly with SCFAs, which were involved in mental function. Dopamine, GABA and serotonin with five, four and three edges, respectively, are other substances that seem to be the main intermediates in this network (**Figure 4**).

### 3.2 Population correlations

**Figure 5:**
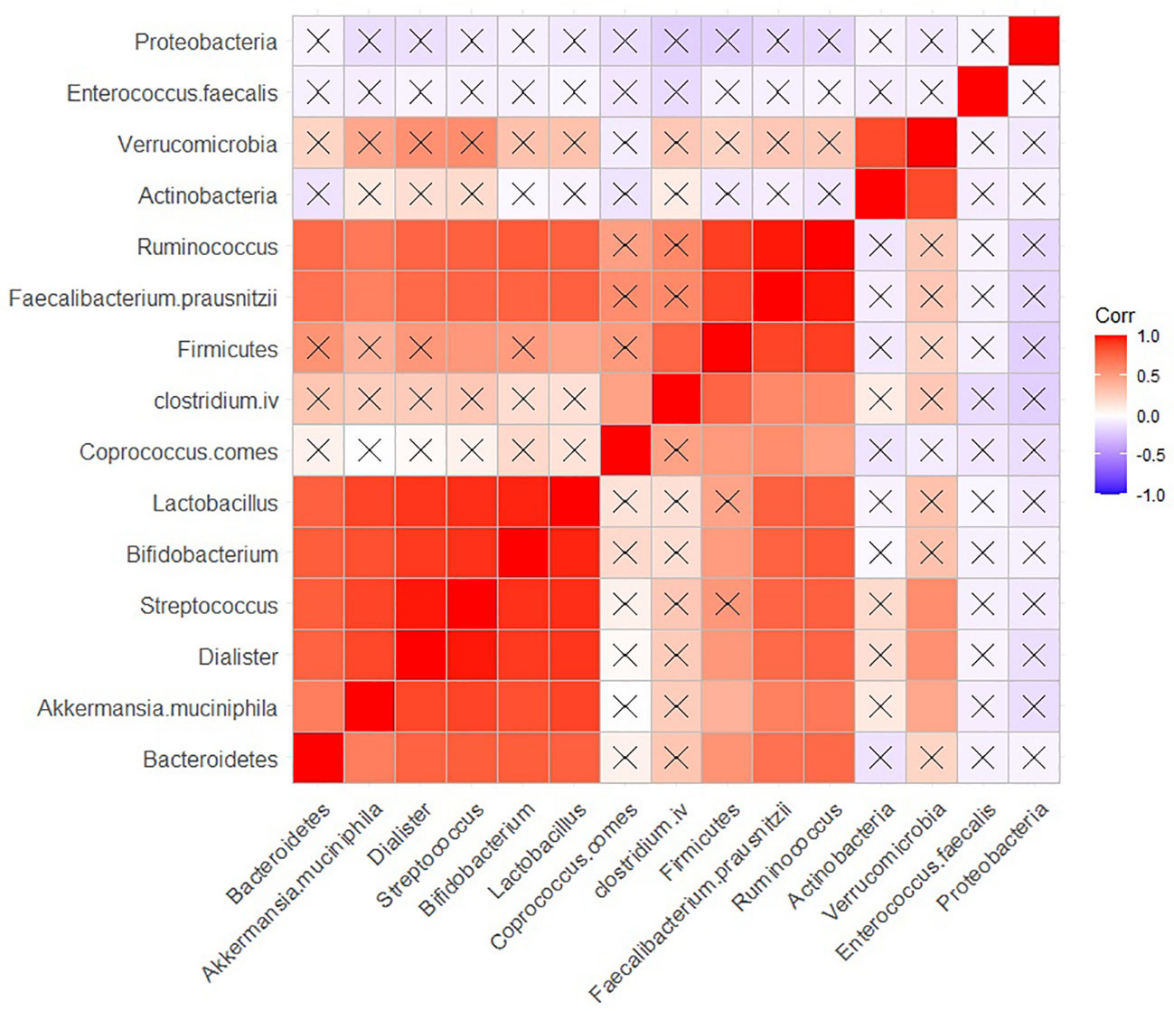
Correlation between the population of different bacteria in BP group. Raw and adjusted probability values are reported below and above the diagonal elements, respectively.

The relationships between bacterial populations in BPs revealed positive significant (adjusted *P* < 0.05) correlations among several species, including *Lactobacillus*, *Bifidobacterium*, *Streptococcus*, *Dialister*, *Akkermansia muciniphila*, *Bacteroidetes*, *Ruminococcus*, *Faecalibacterium prausnitzii* and *Ruminococcus* (**Figure 5**). *Faecalibacterium prausnitzii* and *Ruminococcus* exhibited positive significant correlations with *Firmicutes* as well. *Firmicutes* showed similar correlation with *Streptococcus* and *Lactobacillus*. Additionally, *Verrumicrobia* demonstrated a positive correlation with *Actinobacteria* (**Figure 5**).

**Figure 6:**
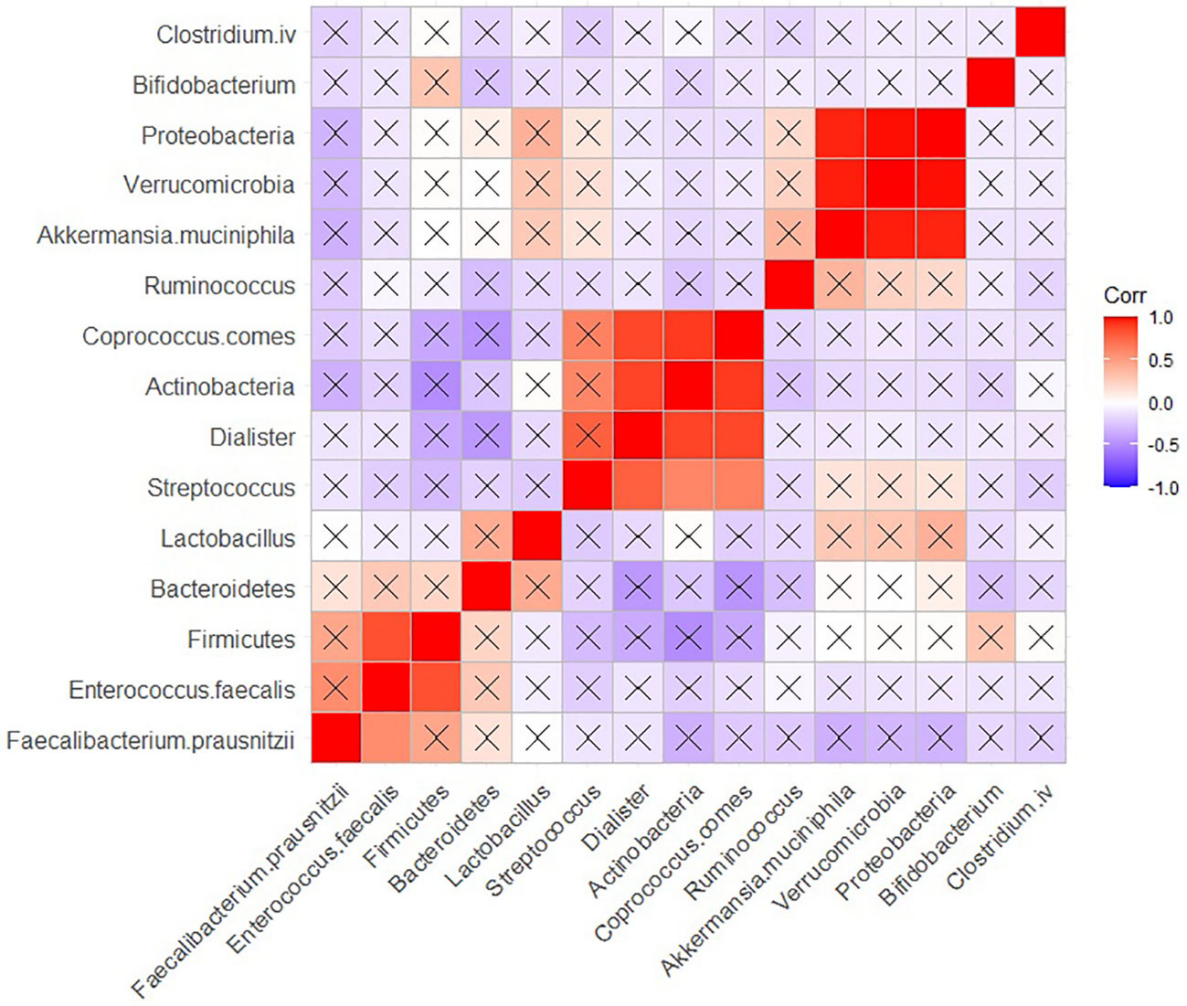
Correlation heatmap of the population of the bacteria in HC group. Raw and adjusted probability values are reported below and above the diagonal elements, respectively.

In HCs, positive, and significant (adjusted *P* < 0.05) correlation was observed between the population of *Proteobacteria*, *Verrucomicrobia*, and *Akkermansia muciniphila*. Furthermore, positive and significant correlations were found between the populations of *Coprococcus comes*, *Actinobacteria* and *Dialister*. Additionally, *Firmicutes* exhibited a positive correlation with *Entrococcus Faecalis* (**Figure 6**).

## 4 Discussion

Dysbiosis in the gut microbiota has been linked to the development of some neuropsychiatric disorders. In the present study, we provided the first evidence for the altered gut microbiota in BPs in the Iranian population. For the selection of samples, caution was taken not to include patients who used certain drugs, had a disease, or had other factors that could affect the bacterial flora. The patients were all hospitalized, and their disorder was confirmed based on an interview and the Diagnostic and Statistical Manual of Mental Disorders (DSM-5) results. Bacteria with a more pronounced impact and change in psychiatric patients were carefully selected for this study.

Our results showed that the genus/species *Clostridium* IV, *Enterococcus faecalis*, *Lactobacillus* and *Coprococcus comes*, phylum *Actinobacteria* and *Proteobacteria* increased and the genus/species*, Bifidobacterium, Dialister, Faecalibacterium prausnitzii*, *Streptococcus, Ruminococcus, Akkermansia muciniphila*, phylum *Verrucomicrobia, Bacteroidetes*, and *Firmicutes* decreased in the stool of BPs compared to HCs showing distinct and altered gut microbiota in BPs.

The gut microbiota produce neurotransmitter such as GABA, serotonin and dopamine which can be released into the blood stream and potentially modulate mental health via the gut-brain axis.

The neurotransmitters GABA, serotonin, and dopamine, known for their crucial roles in the nervous system, have sparked interest in their potential effects on the immune system (Herr et al., 2017; Matt and Gaskill, 2020; Bhandage and Barragan, 2021; Oshaghi et al., 2023). Serotonin and dopamine receptors found on immune cells imply modulatory effects on them (Herr et al., 2017; Channer et al., 2023). Additionally, certain antidepressant medications regulating neurotransmitters exhibit immunomodulatory properties (Nestler et al., 2002). Nevertheless, the immune system’s core regulation primarily relies on cytokines and immune-specific factors. Serotonin-immune cell interaction has been link to neurobehavioral disorders by modulational inflammatory cytokine acting withing the brain (Baganz and Blakely, 2013). Also, dopamine signaling can modulate cytokine secretion and immune function and provide for immunological behavior-switching (Hodo et al., 2020). The above evidences may lead us to extrapolate that the immune system may play a mediatory role in the gut-brain-axis.

Serotonin, commonly referred to as the “hormone of happiness,” is implicated in the etiology of depression, sadness, apathy, and anxiety when deficient(Góralczyk-Bińkowska et al., 2022). The present results revealed that the abundance of *Streptococcus* and *Lactobacillus* species, as serotonin-producing bacteria, decreased and increased, respectively, in BPs compared to HCs. Previous studies have reported an increase in both bacterial populations in BPs (Lai et al., 2021; Lucidi et al., 2021; McGuinness et al., 2022). Decreased serotonin in the depressive phase of bipolar disorder may be associated with decreased *Streptococcus* (Mahmood and Silverstone, 2001). Additionally, a study has reported a positive correlation between serotonin and *Proteobacteria* (Barandouzi et al., 2022). In our study, *Proteobacteria* was higher in BPs than HCs. Whether *Proteobacteria* is also associated with the level of serotonin in bipolar people needs to be elucidated in the future.

Dopamine is another neurotransmitter that potentially plays a role in individuals with bipolar disorder and its associated dysfunctions (Ashok et al., 2017). Moreover, previous studies have suggested that certain gut bacteria can affect dopamine metabolism and function, including *Lactobacillus*, *Bifidobacterium*, *Ruminococcus*, *Clostridium*, and *Enterococcus faecalis* (González-Arancibia et al., 2019; Hamamah et al., 2022). In this study, we investigated the abundances of these bacteria and found a decrease in *Ruminococcus*, and *Bifidobacterium* an increase in *Lactobacillus*, *Clostridium* IV, and *Enterococcus faecalis* indicating that alterations in these gut bacteria may be linked to bipolar disorder in affected individuals.

GABA serves as the principal inhibitory neurotransmitter within the brain, and is capable of being synthesized by gut bacteria. Geuze et al. showed the association of GABA and mental health in patients with depression and anxiety(Geuze et al., 2008). Regarding GABA neurotransmitter-producing bacteria, our findings showed a decrease in *Bifidobacterium*, and *Streptococcus* species and an increase in the abundance of *Lactobacillus, Proteobacteria* and *Enterococcus faecalis*, which might cause an imbalance in the abundance of GABA-producing bacteria in BPs and impair the normal function of the gut-brain axis. Previous studies have reported an increase in *Lactobacillus, Bifidobacterium, Proteobacteria* and, *Streptococcus* bacteria in BPs (Hu et al., 2019; Lai et al., 2020; Lai et al., 2021; Lucidi et al., 2021; McGuinness et al., 2022).

In the current study, the proportion of SCFA-producing bacteria, including *Bifidobacterium, Firmicutes, Streptococcus, Akkermansia muciniphila, Bacteroidetes, Dialister, Ruminococcus*, and *Faecalibacterium prausnitzii*,was lower abundance and *Proteobacteria*, *Actinobacteria, Clostridium* IV and *Lactobacillus* were higher abundance in BPs, which is consistent with previous research (Hu et al., 2019; Lu et al., 2019; Parada Venegas et al., 2019; Guo et al., 2020; Lai et al., 2020; Markowiak-Kopeć and Śliżewska, 2020; Lucidi et al., 2021; Li Z.et al., 2022). In general, the finding suggests that individuals with bipolar disorder exhibit a decrease in the abundance of bacteria that produce SCFA, which may imply their potential advantageous impact (Knuesel and Mohajeri, 2021). Our study is probably the first report showing a decrease in the abundance of *Coprococcus comes* in BPs compared to HCs. In contrary to the current findings, the abundances of *Bifidobacterium, Streptococcus, Verrucomicrobia*, and *Bacteroidetes* populations were higher in bipolar people in previous studies (Dong et al., 2021; Lucidi et al., 2021; McGuinness et al., 2022). These bacteria are involved in the production of SCFAs and they may affect the human body and mental health and reduce intestinal inflammation, which is very common in BPs. BPs have had lower levels of SCFA compared to HCs (Dai et al., 2022). This could be caused by reduced population of these bacteria in BPs (Rivière et al., 2016; Dalile et al., 2019b; Lu et al., 2019; Parada Venegas et al., 2019; Markowiak-Kopeć and Śliżewska, 2020; Müller et al., 2021). Reduction in the production of SCFA and the bacteria responsible for their synthesis could provide evidence for the correlation between these bacteria and bipolar disorder and would demonstrate a link between gut bacteria and mental health. SCFA produced from intestinal microbiota, in addition to the local effect in digestion, by binding to brain cell receptors, can affect gut-brain signaling (Dalile et al., 2019a). Moreover, short-chain fatty acids (SCFAs) induce neuroendocrine cells to convert amino acids into serotonin (Smith and Wissel, 2019). Therefore, SCFAs could also potentially affect mental health through the action of serotonin.

Similar to the result of the previous study, *Ruminococcus* decreased in bipolar people which may reinforce the idea of impaired energy metabolism in BPs (Hu et al., 2019).

Based on our data, *Verrucomicrobia* decreased in BPs. Dong et al. (Dong et al., 2021) reported an increase in the population of *Verrucomicrobia* in patients with general anxiety disorder, whereas Hemmings et al. (Hemmings et al., 2017) showed a reduction in this population in patients with posttraumatic stress disorder. This could be related to differences between mental illnesses.

In the current study, a relationship was observed between bacteria producing small molecule metabolites. This relationship was pronounced for SCFAs, GABA, dopamine and serotonin. These bacteria with overlapping metabolites could be potentially involved in cognitive impairments in BD. Therefore, our results suggest that imbalances in GABA and SCFAs might be potential metabolites that play a role in malfunctioning of gut-brain axis in bipolar patients.

Regarding the clinical characteristics in the current study, all the assessed blood parameters were in the normal physiological ranges in the both groups, showing the normal body condition of all participants. In addition, the parameters were not significantly different between the groups suggesting that these parameters have not affected the gut-brain axis. However, the level of FT3 was rather controversial and was significantly lower in BPs than HCs (P<0.001). Long-term lithium treatment has been shown to decrease FT3 (Kraszewska et al., 2019). As lithium consumption is a routine treatment for bipolar patients, it may have impacted the blood concentration of FT3 in the BPs.

In the present study, significant differences were found in the microbiota of BP and HC groups.

*Firmicutes* are gram-positive bacteria that can play an essential role in both mental and physical health due to their effects on the production of SCFAs. *Bacteroidetes*, on the other hand, are gram-negative bacteria that can induce cytokine synthesis and strengthen immune reactions through their lipopolysaccharides and flagellin components (Stojanov et al., 2020). Several studies have shown that the F/B ratio is related to health status and plays a significant role in maintaining intestinal homeostasis. Deviations from the F/B ratio seen in healthy controls have been correlated with various diseases (Stojanov et al., 2020; An et al., 2023), and in increases in this ratio have been documented in studies on conditions such as Autism spectrum, irritable bowel syndrome, and obesity (Bahr et al., 2015; Lach et al., 2018; Huang et al., 2019; Stojanov et al., 2020). In our study, we found that the F/B ratio was higher in BPs compared to HCs, suggesting that this parameter could also be considered as a candidate marker for BP.

We conducted a series of correlation studies between the populations of bacteria assessed in this study within the BP and HC groups. No significant negative correlation was observed between the bacterial populations, indicating the absence of a competitive relationship among these bacteria. However, we did find a significant positive correlation between certain bacteria, suggesting a prevalent commensalism and mutualism relationship among them.

In BPs, Lactobacillus, *Bifidobacterium*, *Streptococcus*, *Dialister*, *Akeermansia muciniphila*, *Bacteroidetes*, *Ruminococcus*, and *Faecalibacterium prausnitzii* showed a strong positive correlation with each other. Similarly, *Verrucomicrobia* and *Actinobacteria*, as well as *Firmicutes*, *Faecalibacterium prausnitzii* and *Ruminococcus* exhibited positive significant correlations with each other. All aforementioned bacteria are SCFA-producing bacteria, which suggests that short-chain fatty acids (SCFAs) may serve as intermediary substances influencing the interactions and population dynamics of these bacteria in BPs.

Furthermore, both *Lactobacillus* and *Bifidobacterium* are among the bacteria that produce GABA and dopamine. This may indicate the presence of potential intermediates that could link these two species together. Future studies should explore these relationships more comprehensively.

In HCs, we found significant positive correlations between *Coprococcus comes*, *Actinobacteria* and *Dialister*. Additionally, a positive correlation was observed between *Proteobacteria*, *Verrucomicrobia*, and *Akkermansia muciniphila*. Short chain fatty acids, which are commonly produced by these bacteria, may serve as candidate intermediates mediating their interactions as well. Moreover, we identified a positive and significant correlation between *Firmicutes* and *Enterococcus faecalis*. In the literature, we could not find any common substances associated with mental functions produced by these bacteria, leaving us unable to speculate about potential intermediates connecting these two bacterial species.

Based on the correlation results, it seems that there is a significant shift in the relationship between bacterial populations in individuals with bipolar disorder compared to healthy controls. This suggests that the level of interactions between bacterial populations increases in BPs due to the higher number of bacteria involved in these interactions. Additionally, the profile of interactions appears to differ, with changes in the species and genera that are correlated with each other. This may indicate that one way in which bipolar disorder affects or is affected by the gut-brain axis is through alterations in the interaction patterns of gut microbiota.

Currently, numerous studies focusing on depression, anxiety, autism, schizophrenia, and bipolar disorder indicate that probiotics represent an effective treatment modality for these mental disorders by enhancing gut permeability (Góralczyk-Bińkowska et al., 2022). The effects of probiotic bacteria on bipolar disorder have received limited research attention to date. Nevertheless, some studies have indicated that probiotics could potentially improve mood and cognitive function in individuals with this disorder. Specifically, probiotic supplementation has been shown to enhance mood and alleviate symptoms of anxiety and depression, as well as decrease the rate of rehospitalization (Aizawa et al., 2018; Reininghaus et al., 2018; Genedi et al., 2019). In addition, probiotics have been shown to be effective in various ways, including the production of short-chain fatty acids, regulation of the immune system, and support of intestinal barrier integrity (Góralczyk-Bińkowska et al., 2022). Normalizing the population of gut bacteria in bipolar and making them more similar to healthy people may represent a promising treatment approach for managing bipolar disorder. Thus, utilizing the bacteria assessed in our study, perhaps as probiotics, could be considered for future studies.

Based on the present results, the abundance of beneficial bacteria such as *Bifidobacterium* and *Akkermansia muciniphila* significantly reduced in BPs compared to HCs, which indicates the significant effect of gut microbiota on BD through the gut-brain axis. The present results were mostly consistent with the results of previous research, although in some cases, different results were obtained, which could originate from racial, genetic, lifestyle, dietary, and other factors that vary from one country to another and have significant effects on the microbiota in humans.

## 5 Limitations

Our study faced several limitations that should be acknowledged. Firstly, the sample size was small, which was due to the difficulty of recruiting individuals with bipolar disorder because of their specific mental conditions and reluctance to seek medical attention. This made the sampling process lengthy and challenging. Additionally, patients had to meet certain criteria to be included in the study, such as not consuming alcohol in the past month, which was a significant limitation given the unique habits and mental conditions of these patients. We also implemented strict exclusion criteria for the dietary habits of participants in our study using a questionnaire. However, as the participants did not consume a fixed and normalized diet, this could have been a source of variation in the data.

## 6 Conclusion

There has been increasing attention paid to the impact of gut microbiota on various diseases, including mental disorders. The bidirectional relationship between the gut and brain is well-documented in numerous studies conducted across different countries. This study is the first of its kind in an Iranian population, and the results indicate that BPs have a distinct gut ecosystem and likely a malfunctioning gut-brain axis. All bacteria examined in this study showed significant differences between BPs and HCs, suggesting that these bacteria could serve as microbial markers for diagnosing bipolar patients. Normalizing their population could be targeted through probiotic or medical interventions to alleviate the signs and symptoms of BD. Our findings could prove valuable to psychologists in clinical settings, as they may gain a deeper understanding of the physiological factors involved in bipolar disorder. Additionally, these results could encourage psychologists to keep a closer eye on their patients' nutritional intake, medication usage, and gut microbiota profiling. Further research can enhance our understanding of the role of these bacterial populations in the GBA, leading to better decision-making in treating BP.

## Data availability statement

The raw data supporting the conclusions of this article will be made available by the authors, without undue reservation.

## Ethics statement

The studies involving humans were approved by The present study was approved by the Iran National Committee for Ethics in Biomedical Research with ethics code IR.IAU.TNB.REC.1400.119. Informed consent was obtained from all participants before enrollment in the study. The studies were conducted in accordance with the local legislation and institutional requirements. The participants provided their written informed consent to participate in this study. Written informed consent was obtained from the individual(s) for the publication of any potentially identifiable images or data included in this article.

## Author contributions

All listed authors have made significant, direct, and intellectual contributions to this work and have granted their approval for its publication.

## References

[B1] Aizawa E. Tsuji H. Asahara T. Takahashi T. Teraishi T. Yoshida S.. (2018). Bifidobacterium and lactobacillus counts in the gut microbiota of patients with bipolar disorder and healthy controls. Front. Psychiatry 9, 730. doi: 10.3389/fpsyt.2018.00730 30713509 PMC6346636

[B2] Akhtar M. Chen Y. Ma Z. Zhang X. Shi D. Khan J. A.. (2022). Gut microbiota-derived short chain fatty acids are potential mediators in gut inflammation. Anim. Nutr. 8, 350–360. doi: 10.1016/j.aninu.2021.11.005 35510031 PMC9040132

[B3] Alioua S. Abdi A. Fhoula I. Bringel F. Boudabous A. Ouzari I. H. (2016). Diversity of Vaginal Lactic Acid Bacterial Microbiota in 15 Algerian Pregnant Women with and without Bacterial Vaginosis by using Culture Independent Method. J. Clin. Diagn. Res.: JCDR 10 (9), DC23–DC27. doi: 10.7860/JCDR/2016/21621.8546 27790434 PMC5071934

[B4] An J. Kwon H. Kim Y. J. (2023). The firmicutes/bacteroidetes ratio as a risk factor of breast cancer. J. Clin. Med. 12, 2216. doi: 10.3390/jcm12062216 36983217 PMC10052522

[B5] Arabestani M. R. Tahmasebi H. Zeyni B. (2017). Diagnostic value of melting curve analysis based on multiplex-real time PCR in identification of enterococci species. J-Mazand-Univ-Med-Sci 26 (145), 234–247. Available at : http://jmums.mazums.ac.ir/article-1-9503-en.html.

[B6] Ashok A. H. Marques T. R. Jauhar S. Nour M. M. Goodwin G. M. Young A. H.. (2017). The dopamine hypothesis of bipolar affective disorder: the state of the art and implications for treatment. Mol. Psychiatry 22, 666–679. doi: 10.1038/mp.2017.16 28289283 PMC5401767

[B7] Baganz N. L. Blakely R. D. (2013). A dialogue between the immune system and brain, spoken in the language of serotonin. ACS Chem. Neurosci. 4, 48–63. doi: 10.1021/cn300186b 23336044 PMC3547518

[B8] Bahr S. M. Tyler B. C. Wooldridge N. Butcher B. D. Burns T. L. Teesch L. M.. (2015). Use of the second-generation antipsychotic, risperidone, and secondary weight gain are associated with an altered gut microbiota in children. Trans. Psychiatry 5, e652. doi: 10.1038/tp.2015.135 PMC4930121 26440540

[B9] Barandouzi Z. A. Lee J. Del Carmen Rosas M. Chen J. Henderson W. A. Starkweather A. R.. (2022). Associations of neurotransmitters and the gut microbiome with emotional distress in mixed type of irritable bowel syndrome. Sci. Rep. 12, 1648. doi: 10.1038/s41598-022-05756-0 35102266 PMC8803858

[B10] Bhandage A. K. Barragan A. (2021). GABAergic signaling by cells of the immune system: more the rule than the exception. Cell Mol. Life Sci. 78, 5667–5679. doi: 10.1007/s00018-021-03881-z 34152447 PMC8316187

[B11] Bolton D. (2013). What is Mental Disorder?An essay in philosophy, science, and values, International Perspectives in Philosophy & Psychiatry (Oxford, 2008; online edn, Oxford Academic, 1 Feb. 2013). doi: 10.1093/med/9780198565925.001.0001

[B12] Bredholt H. Fjaervik E. Johnsen G. Zotchev S. B. (2008). Actinomycetes from sediments in the Trondheim fjord, Norway: diversity and biological activity. Mar. Drugs 6, 12–24. doi: 10.3390/md6010012 18648671 PMC2474955

[B13] Bromberg-Martin E. S. Matsumoto M. Hikosaka O. (2010). Dopamine in motivational control: rewarding, aversive, and alerting. Neuron 68, 815–834. doi: 10.1016/j.neuron.2010.11.022 21144997 PMC3032992

[B14] Channer B. Matt S. M. Nickoloff-Bybel E. A. Pappa V. Agarwal Y. Wickman J.. (2023). Dopamine, immunity, and disease. Pharmacol. Rev. 75, 62–158. doi: 10.1124/pharmrev.122.000618 36757901 PMC9832385

[B15] Chauvet-Gélinier J. C. Gaubil I. Kaladjian A. Bonin B.. (2012). Trouble bipolaire et comorbidités somatiques : diabète et troubles cardiométaboliques Données physiopathologiques [Bipolar disorders and somatic comorbidities: a focus on metabollic syndrome, diabetes and cardiovascular disease]. Encephale. 38 Suppl 4, S167–72. doi: 10.1016/S0013-7006(12)70095-7 23395232

[B16] Chekole Y. A. Abate S. M. (2021). Global prevalence and determinants of mental health disorders during the COVID-19 pandemic: A systematic review and meta-analysis. Ann. Med. Surg. (2012) 68, 102634–102634. doi: 10.1016/j.amsu.2021.102634 PMC8346529 34386226

[B17] Chen Y.-H. Zhou C.-H. Yu H. Wu W.-J. Wang Y.-W. Liu L.. Gut microbial signatures and differences in bipolar disorder and schizophrenia of emerging adulthood. CNS Neurosci. Ther. 29 Suppl 1(Suppl 1), 5–17. doi: 10.1111/cns.14044 PMC10314106 36468448

[B18] Christensen M. K. Lim C. C. W. Saha S. Plana-Ripoll O. Cannon D. Presley F.. (2020). The cost of mental disorders: a systematic review. Epidemiol. Psychiatr. Sci. 29, e161. doi: 10.1017/S204579602000075X 32807256 PMC7443800

[B19] Clark A. Mach N. (2016). Exercise-induced stress behavior, gut-microbiota-brain axis and diet: a systematic review for athletes. J. Int. Soc. Sports Nutr. 13, 43. doi: 10.1186/s12970-016-0155-6 27924137 PMC5121944

[B20] Collins S. M. Surette M. Bercik P. (2012). The interplay between the intestinal microbiota and the brain. Nat. Rev. Microbiol. 10, 735–742. doi: 10.1038/nrmicro2876 23000955

[B21] Cryan J. F. Dinan T. G. (2012). Mind-altering microorganisms: the impact of the gut microbiota on brain and behaviour. Nat. Rev. Neurosci. 13, 701–712. doi: 10.1038/nrn3346 22968153

[B22] Culpepper L. (2014). The diagnosis and treatment of bipolar disorder: decision-making in primary care. primary Care companion CNS Disord. 16, 13r01609. doi: 10.4088/PCC.13r01609 PMC4195640 25317368

[B23] Dagorn A. Chapalain A. Mijouin L. Hillion M. Duclairoir-Poc C. Chevalier S.. (2013). Effect of GABA, a bacterial metabolite, on Pseudomonas fluorescens surface properties and cytotoxicity. Int. J. Mol. Sci. 14, 12186–12204. doi: 10.3390/ijms140612186 23743829 PMC3709781

[B24] Dai W. Liu J. Qiu Y. Teng Z. Li S. Yuan H.. (2022). Gut microbial dysbiosis and cognitive impairment in bipolar disorder: current evidence. Front. Pharmacol. 13, 893567. doi: 10.3389/fphar.2022.893567 35677440 PMC9168430

[B25] Dalile B. Van Oudenhove L. Vervliet B. Verbeke K. (2019a). The role of short-chain fatty acids in microbiota-gut-brain communication. Nat. Rev. Gastroenterol. Hepatol. 16, 461–478. doi: 10.1038/s41575-019-0157-3 31123355

[B26] Dalile B. Van Oudenhove L. Vervliet B. Verbeke K. (2019b). The role of short-chain fatty acids in microbiota–gut–brain communication. Nat. Rev. Gastroenterol. Hepatol. 16, 461–478. doi: 10.1038/s41575-019-0157-3 31123355

[B27] Demily C. Jacquet P. Marie-Cardine M. (2009). How to differentiate schizophrenia from bipolar disorder using cognitive assessment? Encephale 35, 139–145. doi: 10.1016/j.encep.2008.03.011 19393382

[B28] Demyttenaere K. Bruffaerts R. Posada-Villa J. Gasquet I. Kovess V. Lepine J. P.. (2004). Prevalence, severity, and unmet need for treatment of mental disorders in the World Health Organization World Mental Health Surveys. Jama 291, 2581–2590. doi: 10.1001/jama.291.21.2581 15173149

[B29] Dhakal R. Bajpai V. K. Baek K. H. (2012). Production of gaba (γ - Aminobutyric acid) by microorganisms: a review. Braz. J. Microbiol. 43, 1230–1241. doi: 10.1590/S1517-83822012000400001 24031948 PMC3769009

[B30] Dickerson F. Severance E. Yolken R. (2017). The microbiome, immunity, and schizophrenia and bipolar disorder. Brain behavior Immun. 62, 46–52. doi: 10.1016/j.bbi.2016.12.010 PMC5503102 28003152

[B31] Dinan T. G. Cryan J. F. (2015). The impact of gut microbiota on brain and behaviour: implications for psychiatry. Curr. Opin. Clin. Nutr. Metab. Care 18, 552–558. doi: 10.1097/MCO.0000000000000221 26372511

[B32] Dong Z. Shen X. Hao Y. Li J. Li H. Xu H.. (2021). Gut microbiome: A potential indicator for differential diagnosis of major depressive disorder and general anxiety disorder. Front. Psychiatry 12. doi: 10.3389/fpsyt.2021.651536 PMC8473618 34589003

[B33] Duranti S. Ruiz L. Lugli G. A. Tames H. Milani C. Mancabelli L.. (2020). Bifidobacterium adolescentis as a key member of the human gut microbiota in the production of GABA. Sci. Rep. 10, 14112. doi: 10.1038/s41598-020-70986-z 32839473 PMC7445748

[B34] Fitzgerald C. B. Shkoporov A. N. Sutton T. D. S. Chaplin A. V. Velayudhan V. Ross R. P.. (2018). Comparative analysis of Faecalibacterium prausnitzii genomes shows a high level of genome plasticity and warrants separation into new species-level taxa. BMC Genomics 19, 931–931. doi: 10.1186/s12864-018-5313-6 30547746 PMC6295017

[B35] Flowers S. A. Ward K. M. Clark C. T. (2020). The gut microbiome in bipolar disorder and pharmacotherapy management. Neuropsychobiology 79, 43–49. doi: 10.1159/000504496 31722343

[B36] Galland L. (2014). The gut microbiome and the brain. J. med. Food 17, 1261–1272. doi: 10.1089/jmf.2014.7000 25402818 PMC4259177

[B37] Genedi M. Janmaat I. E. Haarman B. Sommer I. E. C. (2019). Dysregulation of the gut-brain axis in schizophrenia and bipolar disorder: probiotic supplementation as a supportive treatment in psychiatric disorders. Curr. Opin. Psychiatry 32, 185–195. doi: 10.1097/YCO.0000000000000499 30920970

[B38] Geuze E. Berckel B. Lammertsma A. Boellaard R. De Kloet C. Vermetten E.. (2008). Reduced GABAA benzodiazepine receptor binding in veterans with post-traumatic stress disorder. Mol. Psychiatry 13, 74–83, 73. doi: 10.1038/sj.mp.4002054 17667960

[B39] González-Arancibia C. Urrutia-Piñones J. Illanes-González J. Martinez-Pinto J. Sotomayor-Zárate R. Julio-Pieper M.. (2019). Do your gut microbes affect your brain dopamine? Psychopharmacol. (Berl) 236, 1611–1622. doi: 10.1007/s00213-019-05265-5 31098656

[B40] Góralczyk-Bińkowska A. Szmajda-Krygier D. Kozłowska E. (2022). The microbiota&ndash;Gut&ndash;Brain axis in psychiatric disorders. Int. J. Mol. Sci. 23, 11245. doi: 10.3390/ijms231911245 36232548 PMC9570195

[B41] Guo P. Zhang K. Ma X. He P. (2020). Clostridium species as probiotics: potentials and challenges. J. Anim. Sci. Biotechnol. 11, 24. doi: 10.1186/s40104-019-0402-1 32099648 PMC7031906

[B42] Hall-Stoodley L. Hu F. Z. Gieseke A. Nistico L. Nguyen D. Hayes J.. (2006). Direct detection of bacterial biofilms on the middle-ear mucosa of children with chronic otitis media. Jama 296, 202–211. doi: 10.1001/jama.296.2.202 16835426 PMC1885379

[B43] Hamamah S. Aghazarian A. Nazaryan A. Hajnal A. Covasa M. (2022). Role of microbiota-gut-brain axis in regulating dopaminergic signaling. Biomedicines 10 (2), 436. doi: 10.3390/biomedicines10020436 35203645 PMC8962300

[B44] Hemmings S. M. J. Malan-Müller S. Van Den Heuvel L. L. Demmitt B. A. Stanislawski M. A. Smith D. G.. (2017). The microbiome in posttraumatic stress disorder and trauma-exposed controls: an exploratory study. Psychosomatic Med. 79, 936–946. doi: 10.1097/PSY.0000000000000512 PMC5763914 28700459

[B45] Hennekens C. H. Hennekens A. R. Hollar D. Casey D. E. (2005). Schizophrenia and increased risks of cardiovascular disease. Am. Heart J. 150, 1115–1121. doi: 10.1016/j.ahj.2005.02.007 16338246

[B46] Hermann-Bank M. L. Skovgaard K. Stockmarr A. Larsen N. Mølbak L. (2013). The Gut Microbiotassay: a high-throughput qPCR approach combinable with next generation sequencing to study gut microbial diversity. BMC Genomics 14, 788. doi: 10.1186/1471-2164-14-788 24225361 PMC3879714

[B47] Herr N. Bode C. Duerschmied D. (2017). The effects of serotonin in immune cells. Front. Cardiovasc. Med. 4, 48. doi: 10.3389/fcvm.2017.00048 28775986 PMC5517399

[B48] Hodo T. W. De Aquino M. T. P. Shimamoto A. Shanker A. (2020). Critical neurotransmitters in the neuroimmune network. Front. Immunol. 11, 1869. doi: 10.3389/fimmu.2020.01869 32973771 PMC7472989

[B49] Holtz C. (2017). Global health care: issues and policies. Jones & Bartlett Publishers.

[B50] Houtman T. A. Eckermann H. A. Smidt H. De Weerth C. (2022). Gut microbiota and BMI throughout childhood: the role of firmicutes, bacteroidetes, and short-chain fatty acid producers. Sci. Rep. 12, 3140. doi: 10.1038/s41598-022-07176-6 35210542 PMC8873392

[B51] Hu S. Li A. Huang T. Lai J. Li J. Sublette M. E.. (2019). Gut microbiota changes in patients with bipolar depression. Advanced Sci. (Weinheim Baden-Wurttemberg Germany) 6, 1900752. doi: 10.1002/advs.201900752 PMC6662053 31380217

[B52] Huang T.-T. Lai J.-B. Du Y.-L. Xu Y. Ruan L.-M. Hu S.-H. (2019). Current understanding of gut microbiota in mood disorders: an update of human studies. Front. Genet. 10. doi: 10.3389/fgene.2019.00098 PMC6389720 30838027

[B53] Kamada N. Seo S. U. Chen G. Y. Nunez G. (2013). Role of the gut microbiota in immunity and inflammatory disease. Nat. Rev. Immunol. 13, 321–335. doi: 10.1038/nri3430 23618829

[B54] Kang D. W. Adams J. B. Gregory A. C. Borody T. Chittick L. Fasano A.. (2017). Microbiota Transfer Therapy alters gut ecosystem and improves gastrointestinal and autism symptoms: an open-label study. Microbiome 5, 10. doi: 10.1186/s40168-016-0225-7 28122648 PMC5264285

[B55] Kaplan H. I. Sadock B. J. Grebb J. A. Kaplan H. I. (2014). Kaplan and Sadock's synopsis of psychiatry: Behavioral sciences, clinical psychiatry. Williams & Wilkins Co, 1994.

[B56] Kaur H. Bose C. Mande S. S. (2019). Tryptophan metabolism by gut microbiome and gut-brain-axis: an in silico analysis. Front. Neurosci. 13. doi: 10.3389/fnins.2019.01365 PMC6930238 31920519

[B57] Khademi M. Vaziri-Harami R. Shams J. (2021). Prevalence of mental health problems and its associated factors among recovered COVID-19 patients during the pandemic: A single-center study. Front. Psychiatry 12, 602244. doi: 10.3389/fpsyt.2021.602244 33868043 PMC8044784

[B58] Knuesel T. Mohajeri M. H. (2021). The role of the gut microbiota in the development and progression of major depressive and bipolar disorder. Nutrients 14 (1), 37. doi: 10.3390/nu14010037 35010912 PMC8746924

[B59] Koh A. De Vadder F. Kovatcheva-Datchary P. Bäckhed F. (2016). From dietary fiber to host physiology: short-chain fatty acids as key bacterial metabolites. Cell 165, 1332–1345. doi: 10.1016/j.cell.2016.05.041 27259147

[B60] Kraszewska A. Ziemnicka K. Jończyk-Potoczna K. Sowiński J. Rybakowski J. K. (2019). Thyroid structure and function in long-term lithium-treated and lithium-naïve bipolar patients. Hum. Psychopharmacol. 34 (4), e2708. doi: 10.1002/hup.2708 31297898

[B61] Kupfer D. J. (2005). The increasing medical burden in bipolar disorder. JAMA 293, 2528–2530. doi: 10.1001/jama.293.20.2528 15914754

[B62] Kurakawa T. Ogata K. Matsuda K. Tsuji H. Kubota H. Takada T.. (2016). Correction: diversity of intestinal clostridium coccoides group in the Japanese population, as demonstrated by reverse transcription-quantitative PCR. PloS One 11, e0152753. doi: 10.1371/journal.pone.0152753 27019282 PMC4809554

[B63] Lach G. Schellekens H. Dinan T. G. Cryan J. F. (2018). Anxiety, depression, and the microbiome: A role for gut peptides. Neurotherapeutics 15, 36–59. doi: 10.1007/s13311-017-0585-0 29134359 PMC5794698

[B64] Lai J. Jiang J. Zhang P. Xi C. Wu L. Gao X.. (2020). Gut microbial clues to bipolar disorder: State-of-the-art review of current findings and future directions. Clin. Transl. Med. 10, e146. doi: 10.1002/ctm2.146 32898322 PMC7423187

[B65] Lai W.-T. Zhao J. Xu S.-X. Deng W.-F. Xu D. Wang M.-B.. (2021). Shotgun metagenomics reveals both taxonomic and tryptophan pathway differences of gut microbiota in bipolar disorder with current major depressive episode patients. J. Affect. Disord. 278, 311–319. doi: 10.1016/j.jad.2020.09.010 32979562

[B66] Layton A. Mckay L. Williams D. Garrett V. Gentry R. Sayler G. (2006). Development of Bacteroides 16S rRNA gene TaqMan-based real-time PCR assays for estimation of total, human, and bovine fecal pollution in water. Appl. Environ. Microbiol. 72, 4214–4224. doi: 10.1128/AEM.01036-05 16751534 PMC1489674

[B67] Li Z. Lai J. Zhang P. Ding J. Jiang J. Liu C.. (2022). Multi-omics analyses of serum metabolome, gut microbiome and brain function reveal dysregulated microbiota-gut-brain axis in bipolar depression. Mol. Psychiatry. 27(10), 4123–4135. doi: 10.1038/s41380-022-01569-9 35444255

[B68] Li Y. Zhang H. Zheng P. Yang J. Wu J. Huang Y.. (2022). Perturbed gut microbiota is gender-segregated in unipolar and bipolar depression. J. Affect. Disord. 317, 166–175. doi: 10.1016/j.jad.2022.08.027 35987305

[B69] Lichtenstein P. Yip B. H. Bjork C. Pawitan Y. Cannon T. D. Sullivan P. F.. (2009). Common genetic determinants of schizophrenia and bipolar disorder in Swedish families: a population-based study. Lancet 373, 234–239. doi: 10.1016/S0140-6736(09)60072-6 19150704 PMC3879718

[B70] Lomholt L. H. Andersen D. V. Sejrsgaard-Jacobsen C. Ozdemir C. M. Graff C. Schjerning O.. (2019). Mortality rate trends in patients diagnosed with schizophrenia or bipolar disorder: a nationwide study with 20 years of follow-up. Int. J. Bipolar Disord. 7, 6. doi: 10.1186/s40345-018-0140-x 30820700 PMC6395457

[B71] Louis P. Hold G. L. Flint H. J. (2014). The gut microbiota, bacterial metabolites and colorectal cancer. Nat. Rev. Microbiol. 12, 661–672. doi: 10.1038/nrmicro3344 25198138

[B72] Lu Q. Lai J. Lu H. Ng C. Huang T. Zhang H.. (2019). Gut microbiota in bipolar depression and its relationship to brain function: an advanced exploration. Front. Psychiatry 10. doi: 10.3389/fpsyt.2019.00784 PMC6828946 31736803

[B73] Lucidi L. Pettorruso M. Vellante F. Di Carlo F. Ceci F. Santovito M. C.. (2021). Gut microbiota and bipolar disorder: an overview on a novel biomarker for diagnosis and treatment. Int. J. Mol. Sci. 22, 3723. doi: 10.3390/ijms22073723 33918462 PMC8038247

[B74] Magne F. Gotteland M. Gauthier L. Zazueta A. Pesoa S. Navarrete P.. (2020). The firmicutes/bacteroidetes ratio: A relevant marker of gut dysbiosis in obese patients? Nutrients 12 (5), 1474. doi: 10.3390/nu12051474 32438689 PMC7285218

[B75] Mahmood T. Silverstone T. (2001). Serotonin and bipolar disorder. J. Affect. Disord. 66, 1–11. doi: 10.1016/S0165-0327(00)00226-3 11532527

[B76] Mangiola F. Ianiro G. Franceschi F. Fagiuoli S. Gasbarrini G. Gasbarrini A. (2016). Gut microbiota in autism and mood disorders. World J. Gastroenterol. 22, 361–368. doi: 10.3748/wjg.v22.i1.361 26755882 PMC4698498

[B77] Mariat D. Firmesse O. Florence L. Guimarăes V. Sokol H. Dore J.. (2009). The Firmicutes/Bacteroides ratio of the human microbiota changes with age. BMC Microbiol. 9, 123. doi: 10.1186/1471-2180-9-123 19508720 PMC2702274

[B78] Markowiak-Kopeć P. Śliżewska K. (2020). The effect of probiotics on the production of short-chain fatty acids by human intestinal microbiome. Nutrients 12, 1107. doi: 10.3390/nu12041107 32316181 PMC7230973

[B79] Marwaha S. Durrani A. Singh S. (2013). Employment outcomes in people with bipolar disorder: a systematic review. Acta Psychiatr. Scand. 128, 179–193. doi: 10.1111/acps.12087 23379960

[B80] Matt S. M. Gaskill P. J. (2020). Where Is Dopamine and how do Immune Cells See it?: Dopamine-Mediated Immune Cell Function in Health and Disease. J. Neuroimmune Pharmacol. 15, 114–164. doi: 10.1007/s11481-019-09851-4 31077015 PMC6842680

[B81] Mazzoli R. Pessione E. (2016). The neuro-endocrinological role of microbial glutamate and GABA signaling. Front. Microbiol. 7. doi: 10.3389/fmicb.2016.01934 PMC5127831 27965654

[B82] McGuinness A. J. Davis J. A. Dawson S. L. Loughman A. Collier F. O’hely M.. (2022). A systematic review of gut microbiota composition in observational studies of major depressive disorder, bipolar disorder and schizophrenia. Mol. Psychiatry 27 (4), 1920–1935. doi: 10.1038/s41380-022-01456-3 PMC9126816 35194166

[B83] Müller B. Rasmusson A. J. Just D. Jayarathna S. Moazzami A. Novicic Z. K.. (2021). Fecal short-chain fatty acid ratios as related to gastrointestinal and depressive symptoms in young adults. Psychosomatic Med. 83, 693–699. doi: 10.1097/PSY.0000000000000965 PMC8428857 34267089

[B84] Murri M. Leiva I. Gomez-Zumaquero J. M. Tinahones F. J. Cardona F. Soriguer F.. (2013). Gut microbiota in children with type 1 diabetes differs from that in healthy children: a case-control study. BMC Med. 11, 46. doi: 10.1186/1741-7015-11-46 23433344 PMC3621820

[B85] Mutz J. Young A. H. Lewis C. M. (2022). Age-related changes in physiology in individuals with bipolar disorder. J Affect Disord 296, 157–168. doi: 10.1016/j.jad.2021.09.027 34601303

[B86] Nestler E. J. Barrot M. Dileone R. J. Eisch A. J. Gold S. J. Monteggia L. M. (2002). Neurobiology of depression. Neuron 34, 13–25. doi: 10.1016/S0896-6273(02)00653-0 11931738

[B87] Nordentoft M. Wahlbeck K. Hallgren J. Westman J. Osby U. Alinaghizadeh H.. (2013). Excess mortality, causes of death and life expectancy in 270,770 patients with recent onset of mental disorders in Denmark, Finland and Sweden. PloS One 8, e55176. doi: 10.1371/journal.pone.0055176 23372832 PMC3555866

[B88] Oshaghi M. Kourosh-Arami M. Roozbehkia M. (2023). Role of neurotransmitters in immune-mediated inflammatory disorders: a crosstalk between the nervous and immune systems. Neurol. Sci. 44, 99–113. doi: 10.1007/s10072-022-06413-0 36169755

[B89] Painold A. Morkl S. Kashofer K. Halwachs B. Dalkner N. Bengesser S.. (2019). A step ahead: Exploring the gut microbiota in inpatients with bipolar disorder during a depressive episode. Bipolar Disord. 21, 40–49. doi: 10.1111/bdi.12682 30051546 PMC6585963

[B90] Parada Venegas D. de la Fuente M. K. Landskron G. González M. J. Quera R. Dijkstra G.. (2019). Short chain fatty acids (SCFAs)-mediated gut epithelial and immune regulation and its relevance for inflammatory bowel diseases. Front. Immunol. 10, 277. doi: 10.3389/fimmu.2019.00277 30915065 PMC6421268

[B91] Pessione E. (2012). Lactic acid bacteria contribution to gut microbiota complexity: lights and shadows. Front. Cell Infect. Microbiol. 2, 86. doi: 10.3389/fcimb.2012.00086 22919677 PMC3417654

[B92] Qian Y. Yang X. Xu S. Wu C. Qin N. Chen S.-D.. (2018). Detection of microbial 16S rRNA gene in the blood of patients with Parkinson’s disease. Front. Aging Neurosci. 10, 156. doi: 10.3389/fnagi.2018.00156 29881345 PMC5976788

[B93] Reichardt N. Duncan S. H. Young P. Belenguer A. Mcwilliam Leitch C. Scott K. P.. (2014). Phylogenetic distribution of three pathways for propionate production within the human gut microbiota. Isme J. 8, 1323–1335. doi: 10.1038/ismej.2014.14 24553467 PMC4030238

[B94] Reininghaus E. Z. Wetzlmair L. C. Fellendorf F. T. Platzer M. Queissner R. Birner A.. (2018). The impact of probiotic supplements on cognitive parameters in euthymic individuals with bipolar disorder: A pilot study. Neuropsychobiology 8, 1–8. doi: 10.1159/000492537 30227422

[B95] Ríos-Covián D. Ruas-Madiedo P. Margolles A. Gueimonde M. De Los Reyes-Gavilán C. G. Salazar N. (2016). Intestinal short chain fatty acids and their link with diet and human health. Front. Microbiol. 7, 185. doi: 10.3389/fmicb.2016.00185 26925050 PMC4756104

[B96] Rivière A. Selak M. Lantin D. Leroy F. De Vuyst L. (2016). Bifidobacteria and butyrate-producing colon bacteria: importance and strategies for their stimulation in the human gut. Front. Microbiol. 7, 979. doi: 10.3389/fmicb.2016.00979 27446020 PMC4923077

[B97] Rodrigues-Amorim D. Rivera-Baltanas T. Regueiro B. Spuch C. De Las Heras M. E. Vazquez-Noguerol Mendez R.. (2018). The role of the gut microbiota in schizophrenia: Current and future perspectives. World J. Biol. Psychiatry 19, 571–585. doi: 10.1080/15622975.2018.1433878 29383983

[B98] Round J. L. Mazmanian S. K. (2009). The gut microbiota shapes intestinal immune responses during health and disease. Nat. Rev. Immunol. 9, 313–323. doi: 10.1038/nri2515 19343057 PMC4095778

[B99] Saha S. Chant D. Mcgrath J. (2007). A systematic review of mortality in schizophrenia: is the differential mortality gap worsening over time? Arch. Gen. Psychiatry 64, 1123–1131. doi: 10.1001/archpsyc.64.10.1123 17909124

[B100] Sampson T. R. Debelius J. W. Thron T. Janssen S. Shastri G. G. Ilhan Z. E.. (2016). Gut microbiota regulate motor deficits and neuroinflammation in a model of Parkinson’s disease. Cell 167, 1469–1480.e1412. doi: 10.1016/j.cell.2016.11.018 27912057 PMC5718049

[B101] Schneeberger M. Everard A. Gómez-Valadés A. G. Matamoros S. Ramírez S. Delzenne N. M.. (2015). Akkermansia muciniphila inversely correlates with the onset of inflammation, altered adipose tissue metabolism and metabolic disorders during obesity in mice. Sci. Rep. 5, 16643. doi: 10.1038/srep16643 26563823 PMC4643218

[B102] Serpa J. Caiado F. Carvalho T. Torre C. Gonçalves L. G. Casalou C.. (2010). Butyrate-rich colonic microenvironment is a relevant selection factor for metabolically adapted tumor cells. J. Biol. Chem. 285, 39211–39223. doi: 10.1074/jbc.M110.156026 20926374 PMC2998102

[B103] Severance E. G. Prandovszky E. Castiglione J. Yolken R. H. (2015). Gastroenterology issues in schizophrenia: why the gut matters. Curr. Psychiatry Rep. 17, 27–27. doi: 10.1007/s11920-015-0574-0 25773227 PMC4437570

[B104] Silva Y. P. Bernardi A. Frozza R. L. (2020). The role of short-chain fatty acids from gut microbiota in gut-brain communication. Front. Endocrinol. 11, 25. doi: 10.3389/fendo.2020.00025 PMC7005631 32082260

[B105] Smith L. K. Wissel E. F. (2019). Microbes and the mind: how bacteria shape affect, neurological processes, cognition, social relationships, development, and pathology. Perspect. Psychol. Sci. 14, 397–418. doi: 10.1177/1745691618809379 30920916

[B106] Stojanov S. Berlec A. Štrukelj B. (2020). The influence of probiotics on the firmicutes/bacteroidetes ratio in the treatment of obesity and inflammatory bowel disease. Microorganisms 8 (11), 1715. doi: 10.3390/microorganisms8111715 33139627 PMC7692443

[B107] Strandwitz P. (2018). Neurotransmitter modulation by the gut microbiota. Brain Res. 1693, 128–133. doi: 10.1016/j.brainres.2018.03.015 29903615 PMC6005194

[B108] Sun B. Zhou D. Tu J. Lu Z. (2017). Evaluation of the bacterial diversity in the human tongue coating based on genus-specific primers for 16S rRNA sequencing. BioMed. Res. Int. 2017, 8184160–8184160. doi: 10.1155/2017/8184160 28904972 PMC5585543

[B109] Taheri Mirghaed M. Abolghasem Gorji H. Panahi S. (2020). Prevalence of psychiatric disorders in Iran: A systematic review and meta-analysis. Int. J. Prev. Med. 11, 21. doi: 10.4103/ijpvm.IJPVM_510_18 32175061 PMC7050223

[B110] Terry N. Margolis K. G. (2017). Serotonergic mechanisms regulating the GI tract: experimental evidence and therapeutic relevance. Handb. Exp. Pharmacol. 239, 319–342. doi: 10.1007/164_2016_103 28035530 PMC5526216

[B111] Van Snellenberg J. X. De Candia T. (2009). Meta-analytic evidence for familial coaggregation of schizophrenia and bipolar disorder. Arch. Gen. Psychiatry 66, 748–755. doi: 10.1001/archgenpsychiatry.2009.64 19581566

[B112] Vital M. Howe A. C. Tiedje J. M. (2014). Revealing the bacterial butyrate synthesis pathways by analyzing (meta)genomic data. mBio 5, e00889. doi: 10.1128/mBio.00889-14 24757212 PMC3994512

[B113] Weimer P. J. Stevenson D. M. Mertens D. R. Thomas E. E. (2008). Effect of monensin feeding and withdrawal on populations of individual bacterial species in the rumen of lactating dairy cows fed high-starch rations. Appl. Microbiol. Biotechnol. 80, 135–145. doi: 10.1007/s00253-008-1528-9 18535825

[B114] Wilens T. E. Biederman J. Adamson J. J. Henin A. Sgambati S. Gignac M. (2008). Further evidence of an association between adolescent bipolar disorder with smoking and substance use disorders: a controlled study. Drug Alcohol Depend. 95 (3), 188–98. doi: 10.1016/j.drugalcdep.2007.12.016 18343050 PMC2365461

[B115] Yi R. Tan F. Liao W. Wang Q. Mu J. Zhou X.. (2019). Isolation and identification of lactobacillus plantarum HFY05 from natural fermented yak yogurt and its effect on alcoholic liver injury in mice. Microorganisms 7, 530. doi: 10.3390/microorganisms7110530 31694208 PMC6920879

